# Handwriting kinematics during learning to write with the dominant left hand in converted left-handers

**DOI:** 10.1038/s41598-023-28911-7

**Published:** 2023-02-07

**Authors:** Laura Stetter, Johanna Barbara Sattler, Christian Marquardt, Joachim Hermsdörfer

**Affiliations:** 1 Chair of Human Movement Science, Department of Sport and Health Sciences, Technical University of Munich, Georg-Brauchle-Ring 60/62, 80992 Munich, Germany; 2 First German Consulting and Information Center for Left-Handers and Converted Left-Handers, Sendlinger Straße 17, 80331 Munich, Germany; 3 Science & Motion GmbH, Fritz-Lange-Straße 2, 81547 Munich, Germany

**Keywords:** Human behaviour, Learning and memory, Motor control

## Abstract

Converting left-handers to their non-dominant right hand was previously widespread, particularly for handwriting. The present study aimed to explore the extent to which adult, converted left-handers can learn writing with their dominant left hand during a 2-year training program. Eleven converted left-handers participated in the training. Handwriting kinematics were assessed at regular intervals (seven sessions) and compared to those of 11 innate left-handed controls matched for age, gender, and overall handedness score for basic (*Finger*, *Wrist*, *Circle*) and complex (*Sentence*, *Copy*) handwriting tasks. Regarding basic tasks in the training group, we found rapid increases in left and right-hand frequency and no significant differences between both hands at any time point, indicating successful hand transfer. After 24 months, training participants significantly surpassed controls for writing frequency in basic tasks with their left hand. For complex tasks, we identified significant increases in the training groups’ left-hand writing frequency and duration between the first and last session. While training participants’ left-hand writing remained significantly slower than their right-hand writing, statistics confirmed final differences between hands only for the duration of the *Sentence* task. Importantly, left-hand writing in the training group was characterized by lower frequency, lower automaticity, and prolonged duration after 24 months compared to innate left-handers. With training participants’ left-hand writing skills significantly increasing for complex tasks and no final statistically significant differences between hands for frequency and automaticity, the program was considered effective. Nevertheless, within 2 years, training participants did not reach innate left-handers handwriting proficiency for complex tasks. Underlying reasons may be various, such as a non-optimal training program, a sensitive period for learning to write, irreversible neural changes during conversion in childhood, age-related decline of motor learning capacity, or retrograde interference between right- and left-hand writing.

## Introduction

Handwriting is perhaps the most elaborate fine motor skill learned during normal development and education. In this respect, handwriting differs from the expertise in manual dexterity of specialists like musicians playing an instrument or goldsmiths producing jewelry. Handwriting is a highly complex skill combining cognitive and motor processes and requiring fast, rhythmic and smooth movements^1^. Proficient writers reach a level of automaticity characterized by low demands on attentional control during execution^2^. Starting with drawing and painting, typically during pre-school, children learn to write letters, words, and sentences in the first school years. This process involves intense training and is completed only after several years of practice^3,4^.

Typically, the hand used for handwriting is the most obvious expression of handedness. About 11% of the population are left-handed^5^ and thus favor their left hand for handwriting. However, until a few decades ago, many left-handers learned handwriting with their non-dominant right hand^6,7^. Such conversions were initiated either by children themselves or their social environment and usually occurred during pre-school or the first years of elementary school^8^. As the main motivation, negative connotations and pathologizations of left-handedness were presumed to prompt attempts to convert left-handers to right-handers by switching the dominant hand use from left to right^9,10^. While such attempts were typically unsuccessful and hand dominance remained left for many everyday skills, converted left-handers commonly continued to use the right hand for handwriting^11^.

Kinematic analysis is a highly sensitive tool for investigating the sensorimotor performance of handwriting^12–14^. Comparison of right-hand writing in innate right-handers and adult, converted left-handers using kinematic analysis revealed no difference, suggesting that behaviorally the same level of expertise was reached even though the left-handers had to use their non-dominant hand during learning in early childhood^7,15,16^. Despite the lack of behavioral differences, brain representations related to handwriting differed between innate right-handers and adult converted left-handers^7,15–18^.

Here we report behavioral data of adult converted left-handers who participated in a 2-year training program to learn writing with their dominant left hand. The motivations for this endeavor were manifold and highly individual, ranging from curiosity or desires to strengthen one’s own identity to feelings that writing with the “wrong” hand may be related to disturbances of memory, concentration, or fine motor skills and to changes in emotions and personality. Like the motivations, the participants’ goals varied from being able to write with both hands to switching to the left dominant hand, which they used for many non-writing activities.

Clearly, learning to write with the non-writing hand in adulthood is a challenging task. Considering that learning handwriting in childhood takes about 10 years^3,4^, it is conceivable that the success of a 2-year program may be limited. Furthermore, motor learning ability is known to decrease with age, so learning the same task in adulthood could be inferior to learning earlier in life in terms of learning speed and acquired skill level^19–21^. Finally, there may be a timeframe during childhood optimal for learning a complex task like handwriting. Such sensitive periods can, for example, play a crucial role in acquiring outstanding musical expertise^22,23^.

On the other hand, transferring a well-learned skill from the non-dominant right hand in a left-hander to the dominant left hand seems the best precondition for a successful transfer, particularly if the left hand is permanently used in daily life for complex non-writing tasks. Transfer of an acquired motor competence from one hand to the other can be precise in certain tasks, such as adaptating to a changed environment, although hand transfer is much decreased when motor skills are learned during longer training periods^24–27^.

In a single-case study, we previously investigated the progress of an adult, converted left-handed female over a 1-year training to write with the dominant left hand^28^. The kinematics of her left-hand writing movements progressively converged with those of her right hand; nevertheless, the kinematics of the dominant left hand after 12 months of practice were still inferior to those of the non-dominant right hand before starting the program^28^. Thus, while converting handedness during childhood led to adapted handwriting in adulthood^7,15,16^, training to write with the dominant hand in adulthood did not result in a perfect alignment of writing kinematics within 12 months in a single converted left-hander^28^.

In the current research, we studied handwriting kinematics in a larger sample of converted left-handers throughout a 2-year process of training to write with the dominant left hand. Thereby, we aimed to identify to which proficiency converted left-handers can learn writing with their dominant left hand in adulthood. Firstly, to evaluate the program’s effectiveness, we examined the development of writing movements of the training groups’ left hand in tasks of varying complexities, from simple repetitive pen movements to copying a longer text. Secondly, we investigated the differences between writing with the left and right hand after the 2-year interval. Finally, we compared participants’ left-hand writing after 2 years with a control group consisting of innate, non-converted left-handers. With previous literature identifying early gains and rapid transferability of competence from one hand to the other for lower-complexity movement sequences^25,26^, we hypothesized that for basic finger-, wrist-, and circling movements, the training group will achieve right-hand performance with the left hand within the first phases of training. For complex handwriting tasks, i.e. writing sentences and longer texts, we expected slower increases in left-hand writing proficiency over the training period, given that in our above mentioned single case-study^28^, writing frequency and duration improved rather slowly but continuously within the first 9 to 12 months of training the dominant hand. However, despite this increase, left-hand writing performance remained below the right hand even after 1 year of training^28^. This finding combined with handwriting acquisition being a process that is completed only after several years of training^3,4^, led us to hypothesize that converted left-handers will not achieve right-hand performance on complex tasks after training their left hand for 2 years and that comparison with non-converted left-handers will still reveal differences in handwriting kinematics.

## Materials and methods

### Study design and participants

The present study followed a longitudinal design. The writing kinematics of converted left-handers training to write with their dominant left hand were assessed with a fixed protocol at baseline and over the 2-year training program 3, 6, 9, 12, 18, and 24 months after the beginning of the training. A deviation of up to 10% of the targeted session time interval was deemed acceptable. A group of non-converted left-handers served as control subjects and participated in a single session to compare the training groups' left-hand writing after 24 months with that of non-converted left-handers.

Eleven converted left-handers were recruited through the *First German Consulting and Information Center for Left-handers and Converted Left-handers* in Munich, which they contacted to learn writing with their dominant left hand under professional supervision. Converted left-handers’ eligibility to partake in the training program was screened at baseline. Inclusion criteria comprised the sole use of the non-dominant right hand for writing accompanied by the use of the dominant left hand for other activities, anticipated regular and consistent participation in the training and kinematic assessment, and physical and psychological suitability. Exclusion criteria were neurological diseases such as multiple sclerosis, epilepsy, or severe migraine. Eleven non-converted left-handers matched for age, gender, and overall handedness score were recruited as controls by word of mouth and social media. The study was performed according to the ethical standards laid down in the Declaration of Helsinki, and all participants provided written informed consent. Part of the converted left-handers participated in a former study^16^ conducted at the University of Hamburg. The approval of this study by the Ethics Committee of the University of Hamburg included the protocol for the training for reversion of converted handedness and the informed consent form.

### Interventions and procedures

#### Training program

The 2-year program was designed to train handwriting performance based on ergonomic principles through daily home-based exercises to develop and enhance training participants' left-hand graphomotor skills. At the beginning of the program, the focus was on practicing components of simple writing movements with the left hand; daily tasks included finger exercises (8 min), hatching, scribbling and drawing (3 min) and tracing exercises (10 min). From about the fifth week onwards, writing simple letter combinations (5 min) was additionally incorporated. Finally, after approximately 6 months, training time for finger exercises and tracing exercises was reduced (finger 3 min; tracing 5 min) and copying texts with the dominant left hand (5 min) was introduced. Thus, the daily training time amounted to approximately 20 to 25 min, depending on the stage of the program. During recurring appointments at the counseling center, which were combined with the assessment of handwriting kinematics, tasks were explained, demonstrated, and training participants' progress was reviewed by a specialized occupational therapist or psychotherapist (left-hander-consultant). Shortly after baseline, the correct writing posture and exercises for the first month of training were set during the first consultation. Within 12 months, most commenced writing entire texts with their left hand. Training progress and adherence were discussed with the supervisor at the regular meetings. In addition, participants kept a diary recording their daily training sessions. For a more detailed, German-language description of the program, see^28^.

#### Analysis of handedness

Handedness was determined with a customized handedness questionnaire (Sattler, 2004 abbreviated version) administered to the training group at baseline and controls at their single testing session. Participants were prompted to directly perform or pantomime the use of everyday objects (e.g. spinning top, beads, container, building blocks, broom, pen, ball, cutlery, sharpener, scissors), which were placed centrally in front of each subject to avoid bias in the tendency to use either hand. For all tasks, hand use was observed and documented. From this, a total percentage score was calculated for each participant, indicating handedness, i.e., the proportion of activities in which the left hand was used. For example, if all tasks were executed with the left hand, the handedness score equaled 100%. An additional distinction was made between activities in which the hand used is hardly affected by the social environment, such as brushing teeth, collecting and stringing beads, opening lids, and holding a hand broom, versus activities that may be susceptible to such influences, i.e., handwriting, throwing, painting, using cutlery and scissors (*less imprinted* versus *imprinted* activities). *Less imprinted* activities are considered particularly valuable to determine innate handedness. Imprinting scores were calculated by dividing the number of *imprinted* (*less imprinted*) activities performed with the left hand by the total number of *imprinted* (*less imprinted*) activities. As for handwriting habits, the questionnaire additionally queried whether participants wrote by hand more than 5 min a day and whether they wrote more frequently with a computer, by hand or used both equally as often. Subjects were classified as frequent or infrequent writers, with writing by hand more than 5 min a day on average corresponding to frequent writing.

#### Kinematic analysis of handwriting

Handwriting traces were recorded using a graphics tablet (Intuos IV, Wacom Co., Ltd., Kazo, Japan) and a wireless digitizer pen with a ballpoint refill, and an integrated sensor to measure the force exerted axially on the pen tip. The registration of the pen tip position data was performed with a temporal resolution of 200 Hz, a spatial resolution of 0.05 mm, and enabled the detection of movements with the tip up to 10 mm above the tablet. The writing traces were transferred to and stored on a computer, and handwriting kinematics were subsequently analyzed using specialized software (CSWin, MedCom, Munich, Germany). Handwriting traces were automatically segmented into up- and downstrokes along the y-axis orthogonal to the left–right writing direction. Pen tip velocity along the y-axis was calculated with Kernel filters^12^. Writing characteristics calculated within the scope of this study included writing frequency (Hz, number of up- and downstrokes per second), writing duration (ms, time from the onset of the first to the offset of the last stroke), and degree of automaticity as the number of inversions in velocity per stroke and number of submovements per movement stroke (NIV; for details, see^12,29^).

**Figure 1:**
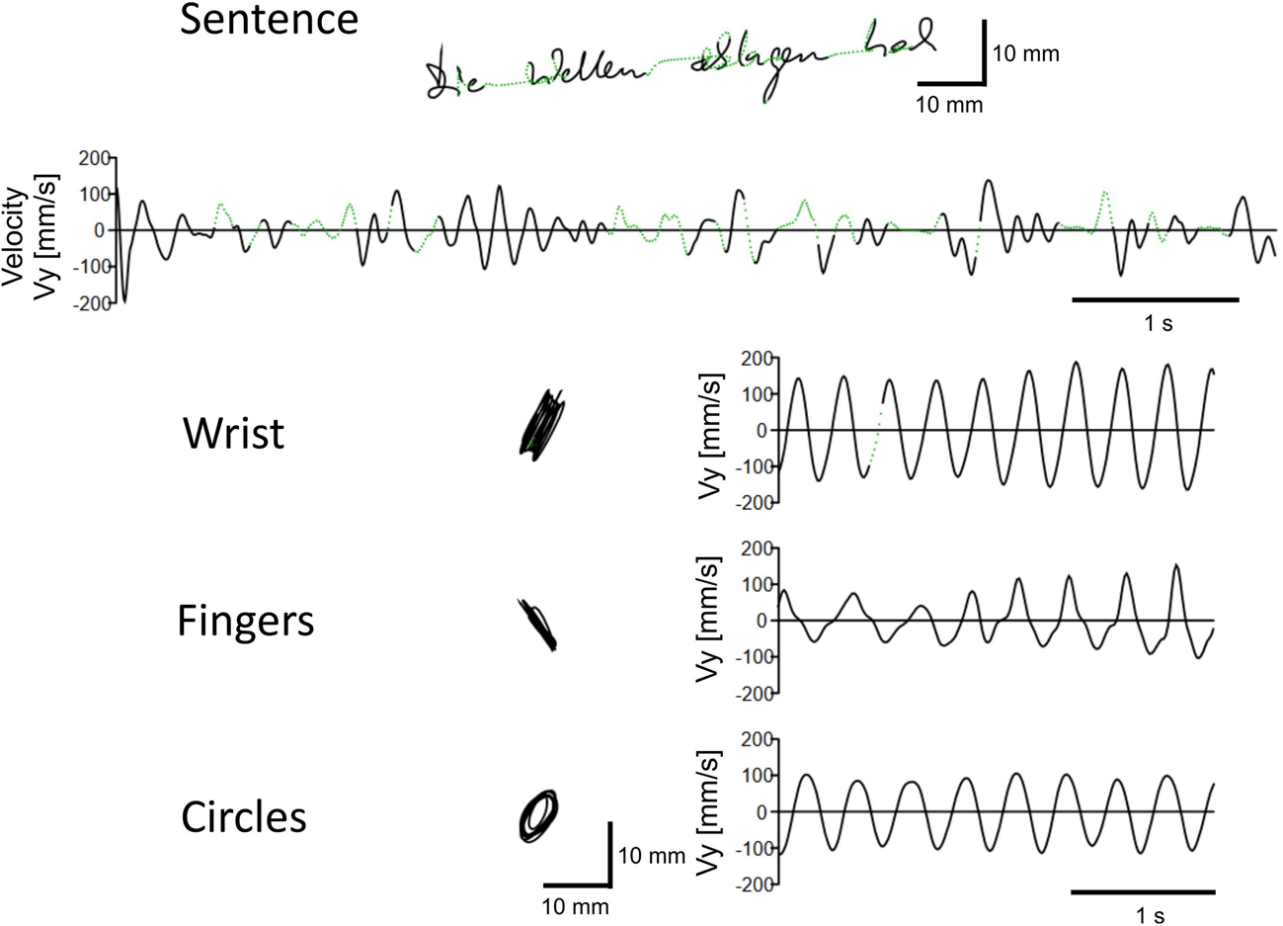
Example scripts and velocity profiles of the handwriting tasks tested in the study. The tasks *Wrist*, *Finger* and *Circle* assess basic aspect of handwriting. The task *Sentence* requests writing of a standard test sentence. This task and a 3-min copy of text (task *Copy*) assess typical and complex aspects of handwriting. Exemplary data shows performance of the right hand of a training participant during the first session following baseline.

The testing procedure included five tasks (see Fig. 1). Repetitive isolated wrist (1—*Wrist* task) and finger flexion–extension (2—*Finger* task) movements with the grasped pen assessed the two basic handwriting elements, i.e. wrist and finger movements, required for performing up- and down strokes, see^1^. The combination of both movements resulted in superimposed circles or “o’s” (3—*Circle* task). The trial duration of the basic handwriting tasks (1–3) was 3 s. Normal handwriting was assessed with writing the German sentence (4—*Sentence* task) “Die Wellen schlagen hoch” (“the waves rise high”). Data recording stopped after the sentence was completed. A longer period of handwriting was tested with a transcription task (5—*Copy* task) in which a text had to be copied as far as possible within a timeframe of 3 min. Participants completed the tasks on a sheet of blank A4 paper positioned directly on the tablet’s surface. Writing samples (*Sentence* and *Copy* task) had to be produced at a normal pace and in individual handwriting without focusing on aspects such as legibility. To support self-chosen writing style and to minimize the influence of speed/accuracy trade-offs, no instructions or indicators were given on font size or shape. The basic writing tasks (*Wrist*, *Finger,* and *Circle* task) were demonstrated before the measurement, and their correct execution was checked. Participants were prompted to perform these tasks fluidly and swiftly. The direction of movements in the *Circle* task could be chosen freely. One trial per task was recorded. In case of errors, repetition of individual tasks was allowed.

Participants always started with the *Sentence* task, followed by the *Copy* task, and then executed the three basic tasks in the order *Wrist*, *Finger,* and *Circle*. In the training group, both hands were tested each session, beginning with the right hand at baseline but with the left hand in all subsequent sessions.

### Data and statistical analysis

Training and control groups were compared regarding age, gender, education, writing habits, handedness scores, and handwriting kinematics. Data on age, gender, education, writing habits and handedness were obtained from the questionnaire for descriptive statistics. As for handwriting habits, information on whether more writing was done by hand or by computer, or equally with both, was missing for one participant in the intervention group. Educational level was categorized as either higher education (academic degree) or vocational training. Between-group differences regarding the categorical variables gender, education and writing habits and the variables age and handedness scores were determined by a Chi-square test and independent-sample t-tests, respectively. In case of lacking normal distribution (Kolmogorov–Smirnov test *p* < 0.05), Mann–Whitney-U tests were used.

Training participants missed a total of seven sessions. Missing sessions were never the first or the last session. Data were complete in six participants; in three participants, one session and in two participants, two sessions were fully missing. For these sessions, missing data were imputed by linear interpolation. Due to technical problems, one training participant additionally had missing data in single tasks of the first session after baseline, with the *Sentence* task missing for both hands and the *Wrist*, *Finger*, and *Circle* tasks missing for the left hand. Since we expected particular learning dynamics at the beginning of the training period, we refrained from imputing these missing data. Consequently, this participant’s data were excluded from the Analysis of Variance (ANOVA, see below), but included in the pairwise tests.

The dependent variable frequency was investigated for all tasks, NIV for the complex handwriting tasks, and duration for the *Sentence* task. Outcome variables were checked for normal distribution (Kolmogorov–Smirnov test *p* > 0.05) for each parameter, group, hand, task, and session.

To examine the effectiveness of training, i.e., the development of the training participants’ left-hand writing over time, and comparison between their right-and left-hand writing, a two-way repeated-measures ANOVA was computed for outcome variables frequency and duration. Thereby, both hand (two levels: left and right) and session (seven levels: baseline, sessions 1–6) were treated as within-subject factors. In the absence of sphericity (Mauchly-test *p* < 0.05), F-test results were reported with a Greenhouse–Geisser correction. For significant interactions, post-hoc comparisons via dependent sample t-tests were carried out to compare the training groups' right- and left-hand performance at each time point and to compare left-hand development in directly consecutive sessions and between baseline and 24 months. To control the overall type I error in multiple comparisons, the significance level of 0.05 was divided by the number of comparisons^30^ for both post-hoc tests, resulting in a significance level of 0.007 each. For the non-normally distributed outcome variable NIV, Friedmann and Wilcoxon tests were utilized to assess training participants' left-hand writing development and to compare both hands at each time point, respectively. Due to multiple comparisons, the significance level for the Wilcoxon test was corrected to 0.007. For significant Friedmann test results, pairwise comparisons of consecutive sessions and between baseline and session 6 after 24 months were carried out using Wilcoxon tests (significance level 0.007).

To compare training participants’ handwriting after 2 years with that of left-handed controls, between-group comparisons were conducted with independent sample t-tests for frequency and duration and Mann–Whitney-U tests for NIV. For heterogeneity of variance (Levene's test *p* < 0.05), t-test results were reported for non-equal variances and corrected for degrees of freedom. To explore a potential relationship between training effects and age, as well as between training effects and the share of imprinted/less imprinted activities performed with the left hand, all participants in the training group were ranked in terms of their increase in left-hand writing performance (greatest improvement—rank 1; lowest improvement—rank 11) between baseline and 24 months for each parameter for the complex handwriting tasks. The aggregate average rank of improvement (lower ranks—greater improvements) resulting for each participant was then correlated with their age, their score for imprinted activities as well as their score for less imprinted activities using Pearson’s r.

Effect sizes applied for significant results were Cohen’s *d* for t-tests, Wilcoxon and Mann–Whitney-U tests, and ${\eta }_{\rho }^{2}$ for ANOVAs. Data analysis was carried out with IBM SPSS Statistics version 28 at an α-level of 0.05.

## Results

### Participants

Twenty-two participants were included in the study; an overview of relevant demographics is provided in Table 1. The training group comprised 11 adult, converted left-handers who switched to their right hand for writing during preschool or the first year of primary school. The control group consisted of 11 innate left-handers without converted handedness. Both groups did not differ from each other regarding the matching variables gender ($\chi$ ^*2*^(1) = 1.64, *p* = 0.395), age (*t*(20) = 0.15,* p* = 0.885), and overall handedness score (*t*(20) = 0.17, *p* = 0.101). Additionally, no group differences were observed for level of education ($\chi$^*2*^(1) = 3.01, *p* = 0.083) and handwriting habits, with seven participants in each group writing more than 5 min per day by hand and five (three/two) participants in the training group and nine (one/two) participants in the control group writing more (less/equally as) frequently by computer than by hand ($\chi$^*2*^(2) = 32.43, *p* = 0.296; see Table 1). For handedness in the imprinted activities, significant and large group differences were found (*U* = 9.00, *p* < 0.001, *d* = 2.13), but not for less-imprinted activities (*U* = 47.50, *p* = 0.401), with training participants using their left hand in just over half (*M* 55.6% *SD* 25.5) of the imprinted activities, on average.

**Table 1:** Comparison of baseline characteristics in training participants and controls.

Baseline characteristic	Training group (n = 11)^a^	Control group (n = 11)^a^	p-value
Age (years)	41.8 (9.3), 27.8–53.6	42.4 (9.9), 23.4–53.6	*p* = 0.885^b^
Gender			*p* = 0.395^c^
Female	4 (36.4%)	7 (63.6%)	
Male	7 (63.6%)	4 (36.4%)	
Level of education			*p* = 0.083^c^
Higher education	4 (36.4%)	9 (81.8%)	
Vocational training	7 (63.6%)	2 (18.2%)	
Writing by hand			*p* = 0.953^c^
≥ 5 min/day	7 (63.6%)	7 (63.6%)	
< 5 min/day	4 (36.4%)	4 (36.4%)	
Computer vs. hand use^d^			*p* = 0.296^c^
Computer	5 (50.0%)	9 (81.8%)	
Hand	3 (30.0%)	1 (9.1%)	
Equal	2 (20.0%)	1 (9.1%)	
Handedness scores (%)			
Overall	71.6 (19.4), 28.9–91.5	84.7 (15.9), 60.0–100.0	*p* = 0.101^b^
Imprinted	55.6 (25.5), 16.7–85.7	89.6 (14.4), 57.1–100.0	*p* < 0.001^e^*
Less imprinted	87.3 (17.6), 39.2–100.0	80.3 (21.0), 37.5–100.0	*p* = 0.401^e^

*p < 0.05 indicating a significant difference between training participants and controls.

^a^Mean (SD), range; n (%); mean (SD), range.

^b^Independent sample t-test.

^c^Chi-square test.

^d^Information missing for one participant in the training group.

^e^Mann-Whitney-U test.

### Basic handwriting skills

**Figure 2:**
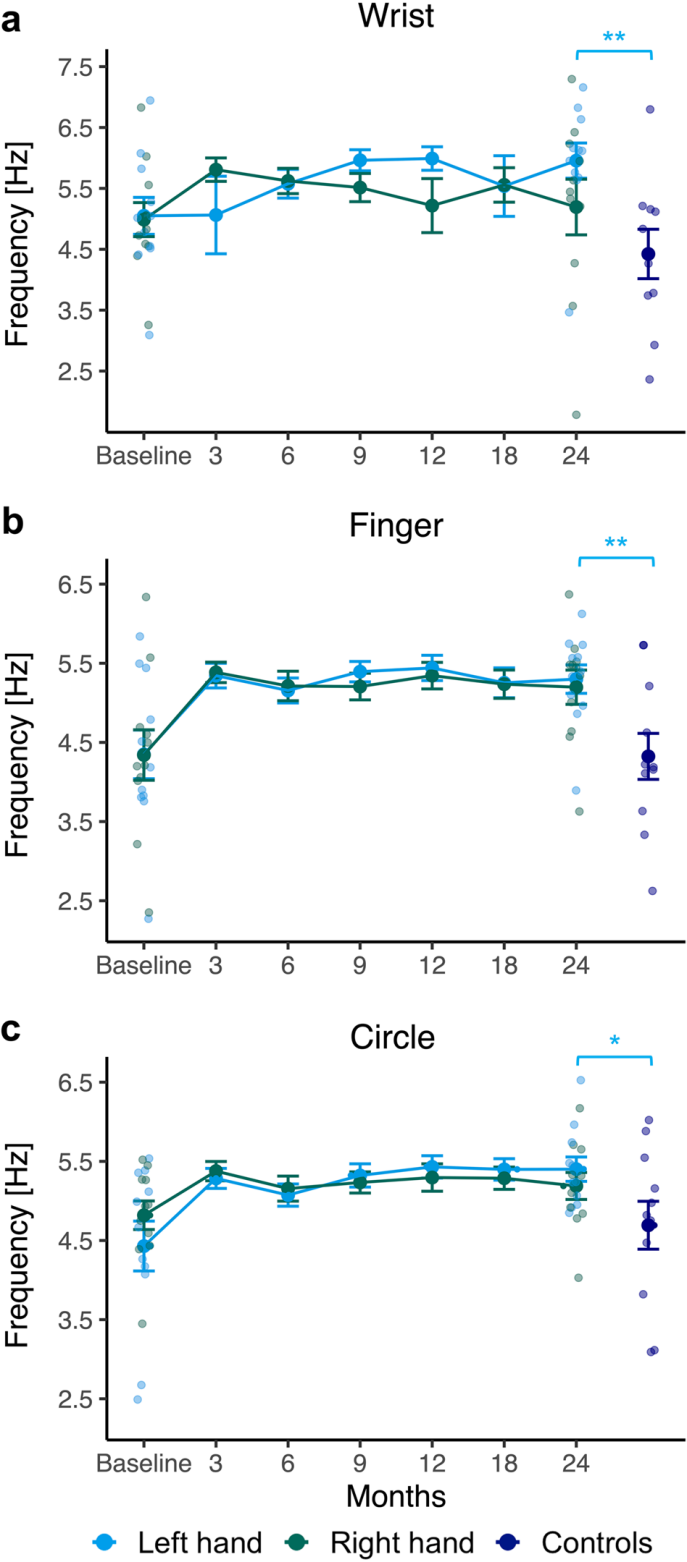
Frequency of pen movements during three basic handwriting tasks ((**a**) *Wrist* task, (**b**) *Finger* task, (**c**) *Circle* task). Line plots show mean and standard errors (SE ± 1) of the results for the dominant left hand (blue) and the non-dominant right hand (green) in converted left-handers across seven sessions during a 2-year training. On the right side, the data of the control group of non-converted left-handers (navy) assessed in a single session is shown. To indicate inter-individual variability, individual data points are shown for the baseline and the final performance of training participants as well as for the control group. Blue bars represent the comparison of training participants' left-hand movements at 24 months vs. controls’ left-hand movements. *p < .05; **p < .01.

Figure 2 shows the time courses of the frequencies across the sessions for both hands in the converted left-handers and the dominant left hand for the non-converted control group for the three basic writing tasks.

For the frequency of the training groups’ movements in the *Wrist* task (Fig. 2a), there were neither significant interactions between hand and session, *F*(2.50, 22.51) = 1.47, *p* = 0.252 nor significant main effects for hand, *F*(1, 9) = 0.03, *p* = 0.857 or session, *F*(6, 54) = 0.97, *p* = 0.456. Compared to controls, training participants performed the *Wrist* task at a significantly higher average frequency with their left, *t*(19) = 3.12, *p* = 0.006, *d* = 1.36 (see Fig. 2a) but not their right hand, *t*(19) = 1.25, *p* = 0.226 at the end of the program.

For repetitive finger movements (*Finger* task), there was no statistically significant interaction between hand and session, *F*(2.72, 24.47) = 0.46, *p* = 0.692, but a significant main effect of session, *F*(1.82, 16.39) = 5.62, *p* = 0.016, ${\eta }_{\rho }^{2}$= 0.38 for the training participants’ movement frequency. Figure 2b suggests the main effect is mainly due to a frequency increase within the first 3 months. Pairwise comparison of the frequencies in the *Finger* task, however, did not yield significant differences between baseline and 3 months (*p* = 0.015, significance level 0.007), any other consecutive sessions (all *p*’s ≥ 0.132), or between baseline and session 6 (*p* = 0.019) when multiple testing was considered. With no significant main effect for hand, *F*(1, 9) = 0.69, *p* = 0.429, the intervention groups' left- and right-hand writing did not differ in this task, irrespective of time point. When comparing the training participants' right- and left-hand movements in the *Finger* task after 24 months with that of the controls' left hand, significant differences were observed, with training participants performing movements significantly faster with both their left, *t*(20) = 2.86, *p* = 0.010, *d* = 1.22 and right hand, *t*(20) = 2.41, *p* = 0.026, *d* = 1.03 than controls (see Fig. 2b).

As observed for finger movements, the training groups' writing frequency in the *Circle* task (Fig. 2c) showed no significant interaction between hand and session, *F*(2.07, 18.59) = 3.13, *p* = 0.066 and no significant main effect for hand, *F*(1, 9) = 0.10, *p* = 0.757, but a significant main effect for session, *F*(6, 54) = 5.31, *p* < 0.001, ${\eta }_{\rho }^{2}$= 0.37. Nevertheless, pairwise comparisons of training participants’ writing frequency detected no significant differences, neither between consecutive sessions (all *p*’s ≥ 0.025, significance level 0.007), despite the visually (see Fig. 2c) distinct increase in mean frequency between baseline and 3 months, nor in the direct comparison of baseline and 24 months (*p* = 0.011). After 24 months, training participants’ left-hand (*t*(14.86) = 2.09, *p* = 0.050, *d* = 0.89; see Fig. 2c) but not right-hand (*t*(20) = 1.43, *p* = 0.169) circling frequency was significantly higher than that of controls using their dominant left hand.

### Complex handwriting skills

**Figure 3:**
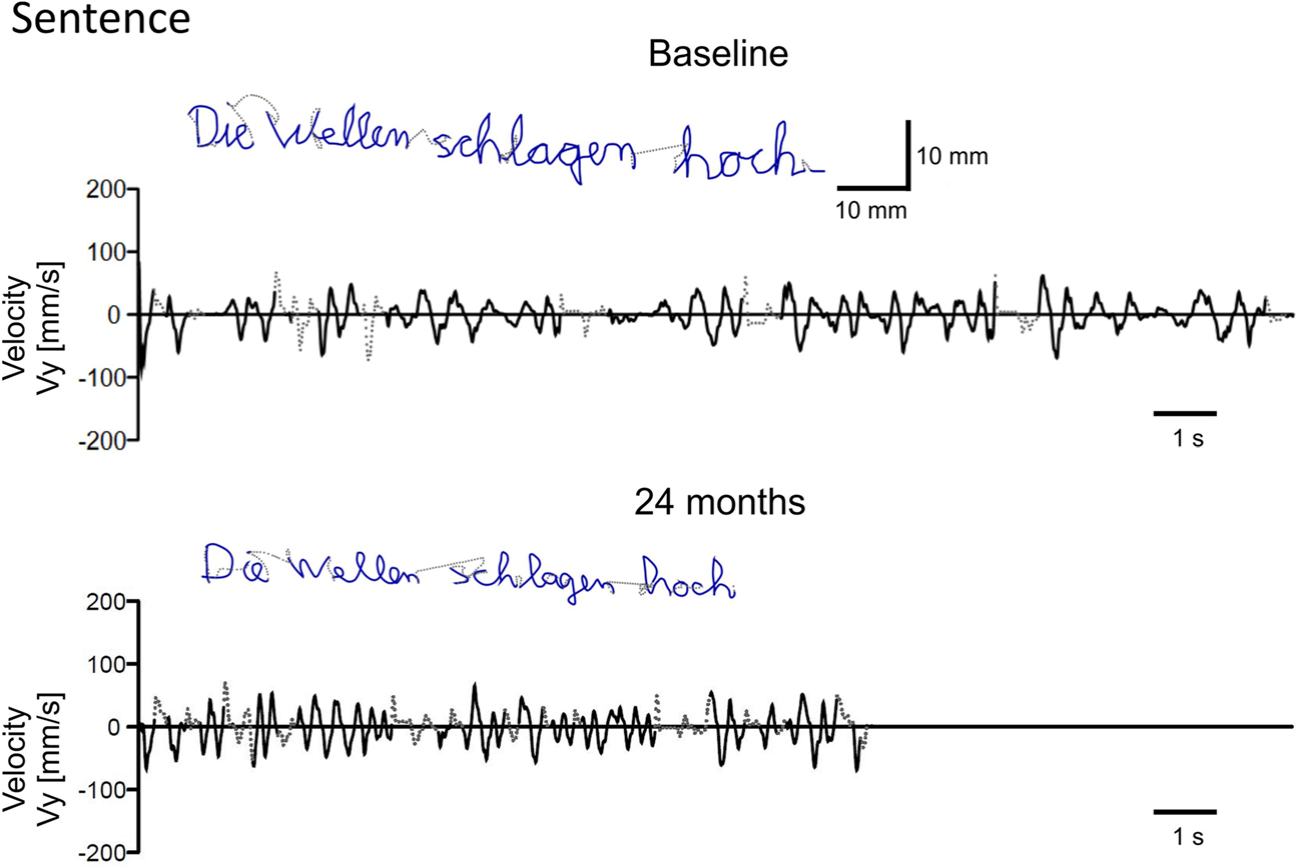
Script and velocity profile during writing the test sentence with the dominant left hand in one training participant at baseline and 24 months after the left-handed participant with converted handedness for handwriting had started a training program to learn writing with his left hand.

Figure 3 shows the script and the velocity profile for performing the *Sentence* task at baseline and in the final session after 2 years of practice for one exemplary training participant. The scripts are relatively similar, besides a more irregular trajectory during the initial writing attempts. Changes are apparent as smoother velocity traces with higher amplitudes after the intervention. In addition, the time needed to write the sentence was substantially shorter at the end of the training (18.26 vs. 11.42 s).

**Figure 4:**
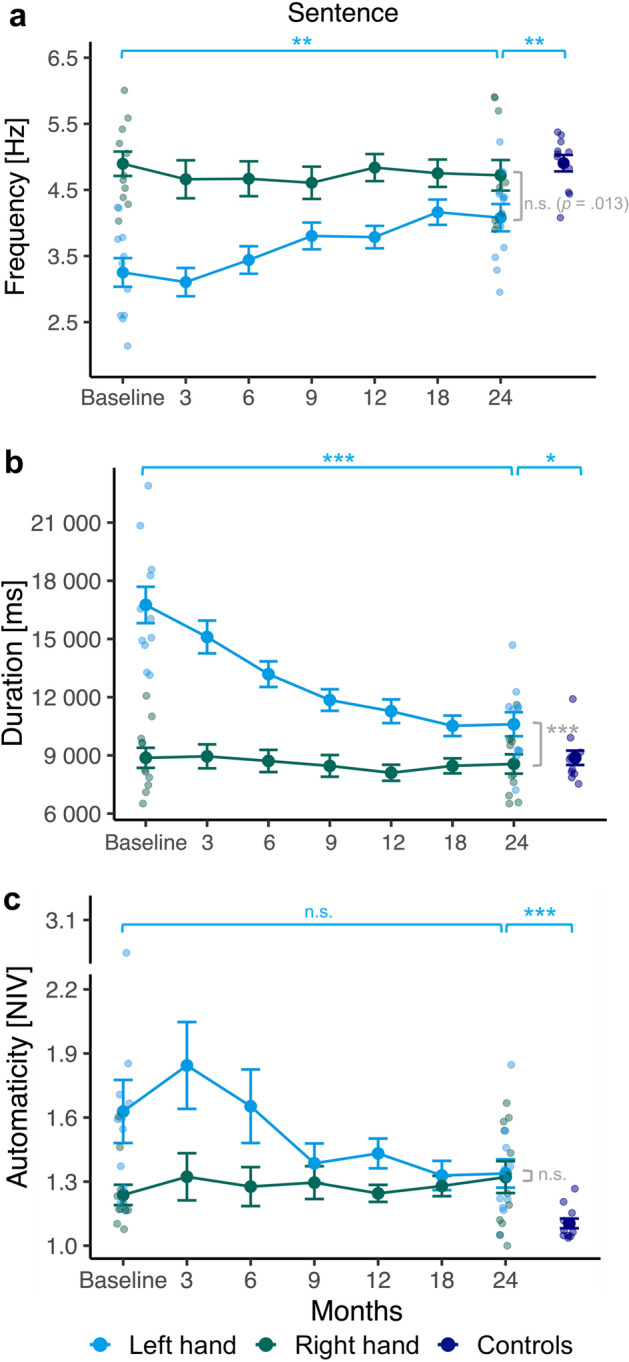
Frequency (**a**), duration (**b**) and automaticity as the number of inversions in velocity (NIV) per stroke (**c**) during the *Sentence* task. Line plots show mean and standard errors (SE ± 1) of the results for the dominant left hand (blue) and the non-dominant right hand (green) in converted left-handers across seven sessions during a 2-year training. On the right side, results from the control group of non-converted left-handers (navy) assessed in a single session are shown. To indicate inter-individual variability, individual data points are shown for the baseline and the final performance of training participants as well as for the control group. Blue bars on the left represent the comparison of the intervention groups’ left-hand movement at baseline vs. at 24 months. Grey bars represent the comparison of training participants' left-hand vs. right-hand movements at 24 months. Blue bars on the right represent the comparison of training participants left-hand movements at 24 months vs. controls’ left-hand movements. *p < .05. **p < .01. ***p < .001.

Figure 4 shows the performance of both groups in the *Sentence* task represented by three parameters. For training participants' handwriting frequency (Fig. 4a), there were significant main effects of hand, *F*(1, 9) = 32.80, *p* < 0.001, ${\eta }_{\rho }^{2}$= 0.79, session, *F*(6, 54) = 4.82, *p* = 0.001, ${\eta }_{\rho }^{2}$= 0.35 and a statistically significant interaction between hand and session, *F*(6, 54) = 9.94, *p* < 0.001, ${\eta }_{\rho }^{2}$= 0.53. As for the development of left-hand writing frequency in directly consecutive sessions throughout the training, significant increases were found between months 6 and 9 (*p* = 0.002). Additionally, the direct comparison between first and last session yielded a large increase in the intervention groups’ left-hand writing frequency for the *Sentence* task (*p* = 0.004; see Fig. 4a). Comparing training participants’ right and left hand at each time point revealed significant differences at baseline, 3, 6 and 12 months (all *p*’s < 0.001). At 9 (*p* = 0.009), 18 (*p* = 0.014), and 24 months (*p* = 0.013), their left-hand performance was not significantly below their right hand (significance level 0.007). Compared with the left-handed control group, the training groups’ left-hand writing frequency in the *Sentence* task was significantly lower after 24 months, *t*(20) = −3.42, *p* = 0.003, *d* = −1.46 (see Fig. 4a), while their right hand did not differ from controls' left hand, *t(*20) = −0.70, *p* = 0.493.

For duration when performing the *Sentence* task (Fig. 4b), the main effects for hand, *F*(1, 9) = 240.02, p < 0.001, ${\eta }_{\rho }^{2}$ = 0.96, session, *F*(1.91, 17.14) = 16.70, p < 0.001, ${\eta }_{\rho }^{2}$ = 0.65 and the interaction between hand and session were also significant, *F*(6, 54) = 29.87, *p* < 0.001, ${\eta }_{\rho }^{2}$ = 0.77 in training participants. Post-hoc pairwise comparisons between consecutive sessions showed significant differences in training participants’ left-hand writing duration between months 3 and 6 (*p* = 0.007) and months 6 and 9 (*p* = 0.002), thereby corroborating the reduction in left-hand writing duration observable in Fig. 4b. A substantial decrease in writing duration was also detected in the direct comparison of their left-hand performance between baseline and 24 months (*p* < 0.001). Nevertheless, the intervention groups’ right-hand writing persisted to be significantly faster than their left-hand writing at each time point (all *p’s* < 0.001). Furthermore, their left-hand writing after 24 months was significantly slower, *t*(20) = 2.40, *p* = 0.026, *d* = 1.02 (see Fig. 4b) than left-hand writing of controls, while duration did not differ, *t*(20) = −0.52, *p* = 0.612 between training participants’ right and controls’ left hand for the *Sentence* task.

As for the development of training participants' left-hand NIV values for the *Sentence* task (Fig. 4c), Friedman tests detected significant differences regarding their mean ranks over all sessions for the left, *χ2*(6) = 13.43, *p* = 0.037, but not the right hand, *χ*2(6) = 1.56, *p* = 0.956. While from Fig. 4c, it seems that the significant effect for the left hand was due to particular improvements in automaticity during months 3 and 9, post-hoc pairwise comparisons via Wilcoxon tests yielded no significant changes in the training groups’ left-hand automaticity, neither between directly consecutive sessions (all *p*’s ≥ 0.014; significance level 0.007) nor between baseline and last session (*p* = 0.067). Their right- and left-hand NIV values significantly differed from each other only at baseline (*p* = 0.002). After 24 months, training participants’ handwriting during the *Sentence* task was characterized by significantly higher mean ranks of NIV for both their left (*U* = 13.50, *p* = 0.001, *d* = 1.75; see Fig. 4c) and right hand (*U* = 28. 00, *p* = 0.034, *d* = 1.02) compared to left-handed controls, indicating an overall less automated handwriting in the intervention group.

**Figure 5:**
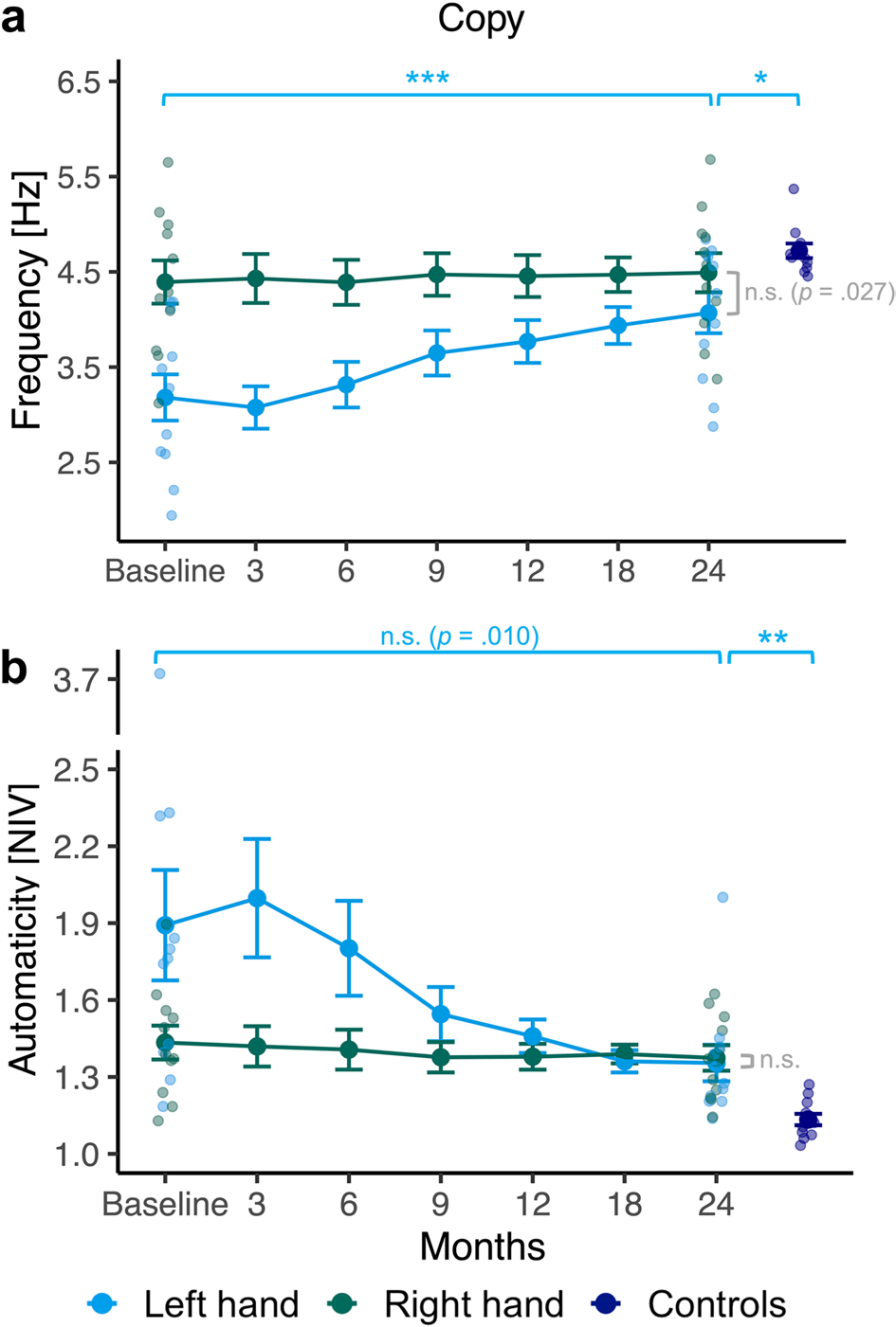
Frequency (**a**) and automaticity as the number of inversions in velocity (NIV) per stroke (**b**) during the *Copy* task. Line plots show results from the intervention group. On the right side, results from the control group are displayed. To indicate inter-individual variability, individual data points are shown for the baseline and the final performance of training participants as well as for the control group. See more explanations in the legend of Fig. 4.

For the *Copy* task (Fig. 5), there were significant main effects for hand, *F*(1, 10) = 22.45, *p* = 0.001, ${\eta }_{\rho }^{2}$= 0.69 and session, *F*(6, 60) = 10.91, *p* < 0.001, ${\eta }_{\rho }^{2}$= 0.52, as well as a statistically significant interaction between hand and session, *F*(6, 60) = 18.57, *p* < 0.001, ${\eta }_{\rho }^{2}$= 0.65 for writing frequency in the training group. Post-hoc pairwise comparisons of their left-hand frequency found significant increases between months 6 and 9 (*p* < 0.001), and between baseline and 24 months (*p* < . 001; see Fig. 5a). As for the comparison of the training groups’ right- and left-hand frequency for the *Copy* task at each time point, post-hoc tests yielded significant differences at baseline, 3, 6, 9, 12 and 18 months (*p*’s ≤ 0.007). After 24 months of training, right- and left-hand frequency did not differ anymore (*p* = 0.027; significance level 0.007). Compared to left-handed controls, training participants’ left- (*t*(12.59) = −2.82, *p* = 0.015, *d* = −1.20; see Fig. 5a), but not right-hand (*t*(12.82) = -1.01, *p* = 0.331) writing frequency was significantly lower for the *Copy* task after 24 months.

The training groups’ NIV pattern for the *Copy* task graphically (Fig. 5b) resembles that for the *Sentence* task (Fig. 4c), albeit at an overall higher, less automated level. Friedman tests to compare training participants' NIV values in the *Copy* task over all sessions produced no significant effects for their right, *χ*^2^(6) = 1.83, *p* = 0.935 but their left hand, *χ*^*2*^(6) = 43.60, *p* < 0.001. Post-hoc pairwise comparisons yielded significant differences only between months 6 and 9 (*p* = 0.001), but not between baseline and 24 months (*p* = 0.010; significance level 0.007). When comparing mean ranks of NIV values between the intervention groups’ right and left hand each session, differences were only significant at 3 months (*p* = 0.005). After 24 months, their left- (*U* = 15.00, *p* = 0.002, *d* = 1.65; see Fig. 5b) and right-hand (*U* = 12.00, *p* = 0.001, *d* = 1.85) performance significantly differed from controls. Consequently, training participants did not attain the degree of automaticity of left-handed controls with either hand for the *Copy* task.

Also of note is the high inter-individual variability, which is evident across all tasks for all parameters, both hands and both groups (see Figs. 2, 4, 5).

The correlation of training effects as the aggregate rank of improvement in complex handwriting tasks (lower ranks—greater improvements) with age yielded a positive r (0.48) with lower ranks in younger training participants but was statistically non-significant (*p* = 0.131). Similarly, training effects did not correlate with either the share of imprinted (r = 0.35, *p* = 0.284) or less imprinted activities (r = 0.43, *p* = 0.184) performed with the left hand, meaning that individual differences in the amount of activities performed with the right hand in addition to handwriting were not predictive of training success.

## Discussion

### Basic handwriting skills

Training participants’ writing frequency for both hands grew over time in the *Finger* and *Circle* task (see Fig. 2), with the greatest improvement within the first 3 months. Additionally, comparisons of the intervention groups’ performance after the program with that of left-handed controls indicated significantly higher frequencies, especially for the left hand. Left-hand improvements can be explained by a generalization of training effects, since writing simple elements constituted an essential part of daily practice, especially during the early training phases. Hand transfer of the required skill may then be responsible for the parallel improvements of the right hand despite absent practice. As outlined in the introduction, hand-transfer may be highly efficient depending on the characteristics of the task. For example, intensive training of tapping movements with the non-dominant hand induced a strong performance increment gain on both hands, which even generalized to similar tasks^31,32^. Alternatively, the rise in frequency might imply learning effects through repeated test administration. However, since only one trial was tested per task and the gain was greatest in the first retest after 3 months, this explanation seems less plausible.

Although the converted left-handers did not use their left hand for handwriting before the training, their basic motor skills did not differ from those of their right hand, even at baseline. Thus, it could be argued that tasks were too easy and that motor dominance might be no critical factor in executing such basic tasks. However, studies on comparable manual motor tasks, such as rapid diadochokinetic finger or wrist (tapping) movements, typically pointed to an advantage of the dominant over the non-dominant hand^33–35^. In accordance with the findings on tapping, Blank and colleagues^36^ identified clear differences in repetitive line and circle drawings very similar to our basic writing tasks, with higher frequencies for the dominant hand, most prominently for small-sized circling movements. In addition to the relative simplicity of the basic tasks, our intervention groups' similar right- and left-hand writing frequencies in the present tasks could be explained by a more balanced hand use in left-handers in general and by the training participants’ converted handedness in particular.

After all, the training group exercised their right hand through decades of handwriting and their left hand through its use in most everyday activities, as indicated by the overall handedness score. Alternatively, less lateralized motor performance was frequently reported in left-handers compared to right-handers^37–39^; therefore, a naturally lower left–right asymmetry in our intervention group may also explain our findings.

### Complex handwriting skills

#### Time course of writing skill acquisition with the left hand

The training successfully increased the performance of the formerly non-writing hand in complex handwriting tasks. At baseline, training participants' left-hand writing performance was worse than that of their right hand, previously used for writing but progressively approached right-hand performance over the training period. Significant interactions between left and right hands’ time courses, significant Friedman tests for NIV values, and significant differences between baseline and 24 months demonstrated highly significant improvements for all parameters and tasks except NIV.

Missing improvements in the pairwise test for NIV in both the *Sentence* and the *Copy* task might be driven by the visible (see Figs. 4c, 5b) but non-significant NIV increase in the training groups’ left-hand writing within the first 3 months, followed by a decrease. Notably, there were also non-significant deteriorations between baseline and 3 months for frequency (see Figs. 4a, 5a). Thus, during the first 3 months of training, handwriting performance seems rather constant or may even be characterized by some deteriorations. As the first program phase encompassed intensive practice of individual movement components, participants may have shifted attention to the accuracy of single components, which has previously been suggested to impair the development of movement fluency^29^.

The 6 months of training following the 3-month session were highly beneficial in improving handwriting performance. Pairwise comparisons of writing parameters between adjacent sessions demonstrated significant improvements between months 3 and 6 and/or between months 6 and 9, also corresponding with the time courses evident in Figs. 4 and 5. While graphically, there was some increment of average performance until month 18, all measures remained quite constant during the final half-year of training.

#### Right-hand performance during training

During the training, the writing kinematics of the right hand were also regularly assessed, with no statistical or visual (Figs. 4, 5) evidence of a performance change. Thus, writing with the non-dominant hand and (successfully) learning to write with the dominant hand yielded no indications of interference, at least for the right hand, and less practice for the non-dominant hand also caused no deterioration of performance. This underlines the high degree of automaticity and overlearning in handwriting, which makes the skill relatively immune to interference and loss of dexterity. Comparing the (unchanged) writing performance of the training participants’ right hand at the end of training with the left hand of non-converted left-handers revealed no difference in frequency or duration, underscoring the high proficiency, to which left-handers were able to learn writing with their non-dominant hand. Consistently, previous research demonstrated no difference in right-hand writing duration^7^ and frequency^15,16^ of adult left-handers who converted to right-hand writing in childhood compared to innate right-handers. However, different from findings by Siebner and colleagues^7^, NIV was significantly lower in the control than in the intervention group indicating a higher degree of automaticity of the non-converted left-handers in both tasks. With previously reported NIV values^7^ being more concordant with those of our training participants, one might argue that our controls, with an average of 1.10 NIV, exhibited exceptionally automated writing, resulting in significant group differences. Nevertheless, the handwriting automaticity of our controls was only slightly above the norm value of 1.13 NIV for performing the *Sentence* task^29^. Thus, the remaining difference in NIV may be a first indicator of a (moderate) deficit in handwriting kinematics and automaticity in left-handers resulting from learning handwriting with the non-dominant right hand during childhood.

Despite the equivalence—at least for frequency and duration—of converted left-handers writing with their non-dominant right hand and innate left handers writing with their dominant left hand at the behavioral level, previous research demonstrated clear differences in patterns of brain activity and brain structure between innate left- and right- handers as compared to converted left-handers^7,15–17^. For instance, a stronger activity in the brain hemisphere contralateral to the dominant hand has been found in innate right- and left- handers, while converted left-handers showed (a more balanced) activity in both hemispheres when writing by hand^7^. Moreover, some activity patterns seem to be modifiable, whereas others remain constant; as such Klöppel and colleagues^15^ identified use-dependent changes in activation in areas of the executive sensorimotor cortex, i.e. the primary sensorimotor hand area and the caudal dorsal premotor cortex, while activity in other areas such as the inferior parietal cortex and rostrolateral premotor cortex was resistant to changes through converting handedness. A review of preference shift studies by Marcori and colleagues^40^ proposed a ceiling effect for changing innate handedness, which would be in line with the reduced right-hand writing automaticity in our training group. In the context of such a ceiling effect, it remains also noteworthy that converting handedness for writing in childhood did not result in a general shift in innate handedness in favor of the right hand in training participants. Consistently with earlier findings^11^, they still performed more activities with their dominant left hand on average, as indicated by the handedness score.

#### Training achievements with the left hand

The study’s key question was whether, after 2 years of practice with the dominant hand, converted left-handers would achieve a writing performance comparable to that of non-converted left-handers. We found no indication of equivalence; instead, both groups differed significantly for each tested parameter. Despite being innate left-handers with solid basic handwriting skills with both hands, potential left-hand writing experiences prior to conversion, and the theoretical ability to transfer handwriting knowledge from the non-dominant to the dominant hand, training participants could not achieve controls’ handwriting proficiency after 24 months of intensive left-hand practice. In contrast, the right-hand conversion in childhood did not result in clear kinematic deficits compared to left-handed controls, at least not for frequency and duration. Thus, while handwriting can be acquired with the non-dominant hand in childhood and shows the same speed and approximate fluency as writing with the dominant hand, our findings imply that learning complex handwriting skills in adulthood might not be possible to the same extent.

The most obvious explanation could be insufficient practice, either in dose or duration. This seems reasonable, given that learning handwriting up to adult proficiency takes about 10 years in childhood^3,4^. However, we found no indication of ongoing improvements during the last half year of the training, suggesting that maximum performance was reached at the end of the training. Still, this does not entirely exclude the possibility that the dose was insufficient or that performance may improve after even longer periods of continuing practice, particularly if individual subjects are considered. Alternatively, handwriting development may be subject to sensitive periods, i.e., time-limited developmental phases during which experiences stimulate neuroplasticity and lastingly affect brain circuits and behavior^41–43^ and therefore have to occur within a specific timeframe to reach their full potential. While sensitive periods have been proposed in visual^44^ and auditory development^45^, language acquisition^46^, musical expertise^22,23^, there is debate about their role in aspects of motor control^47–51^. Residual plasticity after completing a sensitive period^41,52,53^ may have enabled participants to significantly increase their left-hand writing performance throughout the program.

Another explanation could be that converting handedness in childhood resulted in irreversible changes in brain plasticity, thus preventing perfect learning of handwriting with the dominant hand in adulthood. Based on findings of a volumetric decrease in putaminal gray matter in converted left-handers compared to innate left- and right-handers, Klöppel and colleagues^16^ suggested that manipulating innate handedness may negatively affect maturation of the basal ganglia, brain nuclei involved in executing complex movement sequences. Accordingly, the persistent differences could be related to conversion-induced irreversible changes in basal ganglia maturation, which might also be responsible for the moderate deficits in our intervention groups’ right-hand writing automaticity.

Non-optimal training success in our study could also be related to differences in motor learning capacity in young and old age^19–21^. While age per se cannot explain differences between age-matched groups, age-related deficits in motor learning could have prevented the adult, converted left-handers from reaching optimal handwriting performance. The non-significant yet positive correlation between training success and age in our intervention group provides weak evidence for an age effect. However, our participants were younger than those in the typical studies on the effects of aging.

Finally, the remaining capacity of using the right hand for handwriting may have prevented a full-blown proficiency for the dominant left hand. Such proactive interference is well-known, particularly when learning complex sensorimotor tasks^54–56^, and would depend critically on how intensively the converted left-handers continued to use their right hand in daily life. Participants’ reports varied in this respect, with most of them no longer using their right hand for handwriting, suggesting that proactive interference played little or no role.

Overall, the univocal identification of the mechanism preventing training participants from reaching the handwriting ability of their non-converted left-handed peers seems difficult. Rather a combination of mechanisms may be responsible for this central finding.

### Strengths and limitations

Limitations of our study are the lack of an a-priori sample size estimation and the relatively small sample size. However, the overall number of converted left-handers opting to learn writing with their dominant left hand in adulthood who also meet the requirements for doing so may be low. Furthermore, being denied the opportunity to learn handwriting with their dominant hand during childhood, our sample is uniquely suitable for studying handwriting development in adulthood. As converted left-handers approached the consultation center on their initiative, the training group is no representative sample, and our results are likely affected by selection bias. Nevertheless, the 2-year training duration with regular and objective assessments of handwriting kinematics and the inclusion of a control group are definite strengths of the current research. Another limitation related to the long training duration is that the exact training content, length, and regularity of daily sessions could not be precisely controlled. However, from the converted left-handers’ reports during regular meetings and from their diary recordings, it seemed probable that recommendations were followed quite accurately. Further, measurements exclusively cover the development of writing kinematics shortly before and during the training period, with no information about the further individual development of left-hand writing after the end of the program, which prevents any conclusions regarding skill retention.

In terms of gender distribution, although there were potentially limiting differences between training participants and controls (see Table 1), earlier research reported no^57,58^ to minor gender differences^4,59^ for sensorimotor skills in basic and complex handwriting tasks in children and adults. The groups also differed non-significantly in terms of educational level, with a greater share of higher education in the control than the intervention group. This can be explained by many of the controls being recruited within the university setting. Controls also wrote more frequently with a computer in their daily life; while the difference was also not statistically significant, it makes it improbable that controls have more handwriting practice than the intervention group.

Additionally, the time lag in data collection must be acknowledged, with training participants' measurements completed in 2012 and controls recruited and examined between 2019 and 2020. However, comparability was ensured by an identical test setup and a common analysis. Finally, our research lacks a right-handed control group, which would have allowed a direct comparison of converted left-handers' right-hand writing performance with that of innate right-handers. However, previous studies found few^59,60^ to no differences^7^ between right- and left-handers' handwriting kinematics despite slight differences in biomechanics.

## Conclusion

This study investigated to which proficiency 11 converted left-handers learned handwriting with their dominant left hand in adulthood during a 2-year training program. While training participants significantly outperformed 11 left-handed controls for basic motor tasks after 24 months, they did not reach innate left-handers handwriting proficiency for complex tasks.

## Data Availability

Due to confidentiality, the data generated and analyzed within this study are not publicly accessible, but will be made available by the corresponding author upon reasonable request.
